# Breast cancer: descriptive profile of 80 women attending breast cancer care in the Department of General and Digestive Surgery of CHU-YO

**DOI:** 10.11604/pamj.2017.28.314.10203

**Published:** 2017-12-26

**Authors:** Hierrhum Aboubacar Bambara, Abdou Azaque Zouré, Alexis Yobi Sawadogo, Abdoul Karim Ouattara, Nabonswindé Lamoussa Marie Ouédraogo, Si Simon Traoré, Youssef Bakri, Jacques Simpore

**Affiliations:** 1 Service of Oncology, University Hospital Yalgado Ouédraogo, Burkina Faso; 2 Biomolecular Research Center Pietro Annigoni (CERBA)/LABIOGENE UFR/SVT, University of Ouaga 1 Pr Joseph Ki Zerbo, Ouagadougou 1, Burkina Faso; 3 Laboratoiry of Biochemistry and Immunology, Faculty of Sciences, University of Mohammed V, Rabat, Morocco; 4 Institute of Health Sciences Research, (IRSS/CNRST)/Department of Biomedical and Public Health, Burkina Faso; 5 Service of Gynecology, University Hospital Yalgado Ouédraogo, Burkina Faso; 6 Service of General Surgery and Digestive, University Hospital Yalgado Ouédraogo, Burkina Faso

**Keywords:** Breast cancer, descriptive profile, risk factors, Burkina Faso

## Abstract

**Introduction:**

Breast cancer is a common cause of death among women in Burkina Faso. The aim of this study was to determine a descriptive profile of 80 women and establish a description of risk factors associated with breast cancer in these women.

**Methods:**

This cross-sectional study recruited women with breast cancer in Ouagadougou. Teaching Hospital Yalgado Ouedraogo in Burkina Faso from January 2015 to February 2016. We have collected data on socio-demographic characteristics, reproductive status, clinical information, treatment and molecular characteristics.

**Results:**

The average age of the study population was 48.2±12.4 years. Family history of breast cancer was reported in 18.75% of the studied participants against 16.25% family history for other types of cancer. Patients from urban areas represented 87.5% of our studied population with 58.75% of household, multiparous (55.0%), no aborts status (56.2%), post-menopausal women (53.75%), no oral contraception (63.75%), regular menstrual cycle (71.25%) and the prevalence of obesity was 12.5%. The clinical and molecular characteristics showed that left-sided breast cancer accounted for 51.25 %, high grade (II and III) represented 93.75 % of cases and the majority of tumors were infiltrating ductal carcinomas (93.75%) with stages III and IV accounted for 50.0%.

**Conclusion:**

This study described the distribution of risks factors in a population of breast cancer women. Although more research are needed to support these findings, a clear understanding of risk factors associated with breast cancer would contribute to significantly reduce breast cancer incidence and mortality in Burkina Faso.

## Introduction

According to GLOBOCAN 2012, breast cancer incidence has increased by more than 25.0% and mortality by 12.9% worldwide. Breast cancer is the most common cause of cancer death among women (522,000 deaths) worldwide. It now represents one in four of all cancers in women [1,2]. Breast cancer is the most commonly diagnosed cancer in Africa and in Sub-Saharan Africa. This cancer also represents the leading cause of death from cancer (63,100 deaths in 2012) [3]. In Burkina Faso during the period from 2012 to 2015, the number of new expected cancer cases was respectively 1,144 and 1,200 with breast cancer. The incidence is 18.1% and it is the most frequent cancer in women [4]. The actual causes of breast cancer are still unclear but several studies have involved a wide variety of factors like age, gender, heredity, reproductive status, diet, carcinoma of the uterus, carcinoma of the ovary anthropometric characteristics, psychological factors and environmental factors as possible etiological factors [5,6]. The high morbidity and mortality associated with female breast cancer in Burkina Faso is very disturbing. Because of late detection and diagnosis of the disease, this scenario also occurs in other developing countries [7]. However, breast cancer remains one of the most preventable and manageable cancers with the improved understanding of the etiology and predisposing risk factors in specific geographical areas [8]. The present study was aimed at determining a reality of the risk factors distribution, associated with breast cancer among women in Ouagadougou Teaching Hospital Yalgado OUEDRAOGO (CHU-YO) in Burkina Faso.

## Methods

### Setting and type of study

This was a cross-sectional study in which breast cancer patients were recruited from January 2015 to February 2016 at their clinic presentation or monitoring of their disease, after obtaining informed consent. Histological confirmation of breast cancer by pathologists was a required criterion for patient inclusion in the study. We included in the study 80 women treated for breast cancer at the Department of General and Digestive Surgery of Teaching Hospital Yalgado Ouedraogo (CHU-YO) in Ouagadougou, Burkina Faso, who consented to participate in the study. A data collection form was used for socio-demographic, clinical and para-clinical characteristics. Patients who gave informed consent were selected from patients diagnosed with breast cancer. All patients were asked to provide detailed information regarding personal and family history of cancer by interview. Clinical and pathological characteristics including age at diagnosis, mono- or bilateral tumor location, marital status, histology, stage of disease, tumour grading were collected from medical records and pathology reports .

### Study population

The population study consisted of 80 women aged 28-80 years with breast cancer, attending the Department of General and Digestive Surgery of CHU-YO. From time of diagnosis to the time of the interview, we have 42 patients diagnosed between 2009-2014, under follow-up, whose medical treatment was finished and 38 patients diagnosed between January 2015 and February 2016, who were still under treatment (surgery or chemotherapy or radiotherapy). A questionnaire was used to collect patient age, stage of disease, histological type, presence/absence of distant metastases, nulliparity, number of pregnancies and children, family history of breast cancer and family history of other type of cancer, menopausal status (pre- or post-menopausal), menarche and the menstrual cycle, oral contraceptive, childbearing, professional status, chemo-, radio- and hormonal treatments received and rural versus urban residence. Patient’s residence was classified as suggested by INSD [9]: urban if the women lived in towns (province capital or urban municipality status) with a population greater than 10,000 inhabitants and rural if the community size was smaller. Obesity was assessed by using the patient height and weight at diagnosis to calculate the body mass index (BMI), obtained by dividing the weight in kilograms by the square of height in meters (kg/m^2^). According to the criteria of the US National Institute of Health/National Heart Lung and Blood Institute (NCI/NHLBI) [**10**], women were classified as obese if BMI was ≥ 30 kg/m^2^, overweight if BMI was between 25 and 30 kg/m^2^ and normal/lean if BMI was < 25 kg/m^2^. The histological grading of the tumors was performed in accordance with the Bloom-Richardson classification (SBR).

### Ethics approval and consent to participate

The whole research was funded by personal resources of researchers. The study was approved by the National Health Ethic Committee of Burkina Faso (reference number 2014-7-085 of July, 7^th^ 2014). All study participants gave their free written and informed consent according to the Helsinki Declarations. Any personal identifier was encoded.

### Statistical analyses

Data obtained were analyzed using Epi Info version 7 and the Statistical Package for the Social Sciences version 17 statistical package (SPSS) Incorporated, Chicago, Illinois, USA.

## Results

Table 1 shows the socio demographic distribution of the study population. The patients’ age range was from 28 to 80 years with the mean of 48.20 years ± 12.4. The majority of the patients in this study (87.50%) were come from the urban area with household (58.75%), married (81.25%), multiparous (55.00%), no aborts status (56.20%), post-menopausal women (53.75%), no oral contraception (63.75%), age of menarche between 12 and 15 years (63.75%), and regular menstrual cycle (71.25%) (Table 1 (suite)). Respondents´ anthropometric indices are presented in Table 1 below. The prevalence of obesity (as measured by BMI) was 12.50% against 26.25% for overweight (Table 1). The relationship between family history and risk of breast cancer is presented in Table 2. A proportion of 18.75% and 16.25% of the study participants reported a family history of breast and other types of cancer respectively. Other cancers have reached mainly lung, eye, skull, stomach… (Table 2). The clinical and molecular characteristics are showed in Table 3. The left-side breast cancer accounted for 51.25% of cases and bilateral breast masses represented 1.25% of cases. The Table 3shows that the high grade (II and III) which constituted 93.75%. The majority of tumors were infiltrating ductal carcinomas (93.75%) followed by other (micropapillary and papillary…) with 6.25% of histological sub-types and infiltrating ductal lobular (1.25%). The presentation of staging (stages I and II) constituted (50.00 %) similarly stages III and IV accounted for (50.00%) (Table 3). Regarding immunohistochemistry, like all clinical and laboratory services, its cost is still borne by patients, or its realization is still rare in Burkina Faso, which makes it inaccessible examination. This accounted were able to realized for n = 11/80; 13.75%). The majority of tumors was luminal A and triple negative, followed by luminal B (Table 4). The treatment-benefited patients are summarized in Table 5. Thus, we have 71.25% who received surgery (mastectomy or lumpectomy), 77.85% have at least been given a chemotherapy session and 16.25% have completed treatment by radiotherapy (Table 5).

**Table 1:** Sociodemographic characteristics and anthropometric indices of respondents

Variable	N = 80 n (%)
**Age**	
≤ 40 years	30 (37.50%)
>40 years	50 (62.50%)
Median ± SD	12.40 years
Range	48.20 years
**Residence**	
Urban	70 (87.50%)
Rural	10 (12.50%)
**Profession**	
Functionaries	24 (30,00%)
Household	47 (58.75%)
Others	9 (11.25%)
**Marital Status**	
Maried	65 (81.25%)
Single/Never maried	9 (11.25%)
Widow	6 (7.50%)
**Body mass index**	
Normal/lean < 25 kg/m^2^	49 (61.25%)
Overweight between 25 and 30 kg/m^2^	21 (26.25%)
Obese ≥30 kg/m^2^	10 (12.50%)

**Table 1 (suite):** Socio-demographic characteristics and anthropometric indices of respondents

**Number of pregnancies**	
Nulliparous	5 (6.25%)
Primiparous	3 (3.80%)
Pauciparous	28 (35.00%)
Multiparous	21 (26.25%)
Great Multiparous	23 (28.75%)
**Abortion Status**	
Yes	35 (43.80%)
No	45 (56.20%)
**Menopausal status**	
Pre	37 (46.25%)
Post	43 (53.75%)
**Oral contraception**	
Yes	29 (36.25%)
No	51 (63.75%)
**Age of menarche**	
≤11 years	2 (2.50%)
Between12years et 15 years	51 (63.75%)
>15 years	27(33.75%)
**Menstrual cycles**	
Regular	57 (71.25%)
Irregular	23 (28.75%)

**Table 2:** Family history of breast and other cancers

Variable	N = 80 n (%)
**Family history of breast cancer**	
Yes	15 (18.75%)
No	65 (81.25%)
**Family history of other cancer type**	
Yes	13 (16.25%)
No	67 (83.75%)

**Table 3:** Clinical and molecular characteristics

Variable	N = 80 n (%)
**Side of Breast**	
Left Breast	38 (47.50%)
Right Breast	41 (51.25%)
Bilateral	1 (1.25%)
**Grade**	
I	5 (6.25%)
II	56 (70.00%)
III	19 (23.75%)
**Histological type**	
Ductal IDC	75 (93.75%)
Lobular ILC	1(1.25%)
Other	5 (6.25%)
**Stage**	
I	5 (6.25%)
IIA/IIB	35 (43.75%)
IIIA/IIIB	23 (28.75%)
IV	17 (21.25%)

IDC: Infiltrant Ductal Carcinoma; ILC: Infiltrant Lobular Carcinoma

**Table 4:** Immunohistochemical classification of tumors

Tumor subtypes	N = 11 n (%)
Luminal A	
(ER+and/or PR+; HER2-)	4 (36.36%)
Luminal B	
(ER+ and/or PR+; HER2+)	2 (18.18%)
HER2+	
(ER-; PR-; HER2+)	1 (0.90%)
Basal	
(ER-; Her2-)	4(36.36%)
Triple negative	
(ER-; PR-; HER2-)	4 (36.36%)

**Table 5:** Medical treatment received by patients

Type	N = 80 (n = %)
**Surgery**	
Yes	57 (71.25%)
No	23 (28.75%)
**Chemotherapy**	
Yes	63 (77.85%)
No	17 (21.25%)
**Radiotherapy**	
Yes	13 (16.25%)
No	67 (82.85%)
**Hormone therapy**	
Yes	14 (17.5%)
No	66 (82.50%)

## Discussion

The purpose of this study was to identify the distribution of risks factors of breast cancer risk among women in Burkina Faso. We recognize bias generated during this epidemiological approach. There are biases related to quality of care, missing data and the relatively small number of subjects. However, given the difficulties, we believe that despite the relatively small size of the sample, preliminary results are reasonable and encouraging.

### Age

Once considered a disease of older women, breast cancer occurs more in young women. Our average age of 48.20 ± 12.40 years shows no statistically significant difference with that reported by other authors in Mali: 47.70 ± 12 years [11]. However, this average is low compared to other countries such as France, United States and Canada, where the breast cancer is a disease of older women (> 55 years) [5]. This difference could be explained by the youthfulness of the African population in general.

### Location and profession

In this study, we found 87.50% of urban area resident women and 58.75% housewives. What is known of the environment - cancer relationship is that cities are increasingly polluted. It has not been established a relationship between the occurrence of cancer and housewife profession unless these women use carcinogens in the kitchen or other places of the house [12].

### Parity

Nulliparity is considered a risk factor for breast cancer and the disease seems strongly linked to marital status. The risk increase with wedding after 30 years of age and in the case of celibacy. Our findings about the relative risk of nulliparity are confirmed by several studies that demonstrate the protective role of multiparity [12]. Unfortunately, women lose this protection after menopause that they are more at risk of developing breast cancer. Our proportion of 55.00% of multiparous is similar to those of Sano et *al.* who found 54.00% of multiparous among women with breast cancer in Burkina Faso [13].

### Oral contraceptives

In our study, we found 36.25 % who practiced oral contraceptives before their breast cancer. This could pose a risk of cancer apparition in our population. The risk of breast cancer is increased by around 25.00% in current users of combined oral contraceptives. However, use of oral contraceptives late in a woman’s reproductive life will result in an increased relative risk of breast cancer at a time when the background risk is becoming appreciable [5].

### Menarche and the menstrual cycle

The older a woman is when she begins menstruating, the lower her risk of breast cancer is. We found 36.25 % of women who had their first menstrual before the age of 11 years or after 15 years of age, which is a breast cancer risk. Our results confirm those reported by Senhadji et *al.* 2010 in Algeria [14]. Nevertheless, irregular menstrual cycles represent 28.75% of our population study. Thus, this hormonal dysfunction could be one of the major factors of breast cancer in our population.

### Anthropometry

Adult height shows a weak positive association with breast cancer risk. Average height is substantially greater in populations with high rates of breast cancer than in populations with low rates. A few studies have reported that breast-cancer risk is increased in women whose birth weight was high [5]. In Nigeria, a study found an inverse relationship between BMI and breast cancer risk. A cohort study in African Americans also showed that a high BMI was associated with reduced risk of breast cancer [7]. This can be explained by the roundness of African women.

### Family history

Most studies on familial risks of breast cancer have found about two-fold relative risks for first-degree relatives (mothers, sisters, daughters) of affected patients. With affected second-degree relatives (grandmothers, aunts, granddaughters), there is a lesser increase in risk [5]. We found 35.00% of patients who have at least one member of their family already affected by breast cancer or other types of cancer. This might explain a hereditary cancer in our population and the young mean age we found.

### Histology

The infiltrating ductal carcinoma is the most common histological form. This concept appears in the Greek playoffs with 42.00%, 90.00% with French, Spanish 82.00%, with Malian 57.14 % and in our series with 93.75% [12].

### Staging

Breast cancer treatment is less aggressive and more effective if diagnosed early. This fact is especially observed in the French and Tunisian series [15] where stage I or II is represented in 73.00% to 74.00%, unlike our Burkinabe series in which the highest proportions are found in stage II or IV, ranging from 43.75% to 21.25%. Hormone receptors are of prognostic and predictive factors essential to the therapeutic management of breast cancer. Thus, in our study only 11 patients were able to perform the review, because the costs are their costs. This situation does not favor the full support of the patient to avoid any recurrence.

### Treatment

Late diagnosis, lack of radiation therapy in Burkina Faso and the difficulties associated with chemotherapy are the reasons for low proportions of patients receiving chemotherapy (77.85%), radiotherapy (16.25%), surgery (71.25%) and hormone therapy (17.50%). The high prices of cancer drugs must endure by patients in the absence of reimbursement system. The access of patients and their families in prevention, diagnosis, treatment and psychosocial support is severely hampered in Africa through the weakness of health systems in terms of governance, financial and human resources and in terms of infrastructure [16].

### Logistic regression

Finally statistical logistic regression analysis of risk factors (age, anthropometric characteristics, family history and reproductive factors) showed none significantly different results. Thus in our population no risk factor can establish with this data. We could have bias given the size our sample and also because the analysis concerned all patients and those with breast cancer. It may be necessary to have a control group compared with our group of patients.

## Conclusion

This study described the distribution of risks factors in a population of breast cancer women. Pending more research and validation, the finding of the relationship between some environmental factors and breast cancer risks, this study could be useful to health authorities in planning interventions to reduce breast cancer incidence and mortality in Burkina Faso. Which brings us to undertake a new study with more power in order to better understand and take a preventive approach.

### What is known about this topic

- Breast cancer is a public health problem in Burkina Faso;
- The risk factors for breast cancer are not yet well understood except for genetic factors;
- In Burkina Faso, as in all of West Africa, there are few studies and data on risk factors for breast cancer; the access of patients to treatment; monitoring and early diagnosis of breast cancer are a crucial problem in Burkina Faso.

### What this study adds

- Our study describes the distribution of known risk factors in a population of women with breast cancer in Burkina Faso;
- We have revealed the diagnostic status and medical treatment received by patients in Burkina Faso.

## Competing interests

The authors declare no competing interests.
